# Full On-Device Stay Points Detection in Smartphones for Location-Based Mobile Applications

**DOI:** 10.3390/s16101693

**Published:** 2016-10-13

**Authors:** Rafael Pérez-Torres, César Torres-Huitzil, Hiram Galeana-Zapién

**Affiliations:** Information Technology Laboratory, CINVESTAV-Tamaulipas, Ciudad Victoria C.P. 87130, Tamaulipas, Mexico; ctorres@tamps.cinvestav.mx (C.T.-H.); hgaleana@tamps.cinvestav.mx (H.G.-Z.)

**Keywords:** stay point, smartphone, Location Based Services (LBS), context-aware, power-aware, event-driven

## Abstract

The tracking of frequently visited places, also known as stay points, is a critical feature in location-aware mobile applications as a way to adapt the information and services provided to smartphones users according to their moving patterns. Location based applications usually employ the GPS receiver along with Wi-Fi hot-spots and cellular cell tower mechanisms for estimating user location. Typically, fine-grained GPS location data are collected by the smartphone and transferred to dedicated servers for trajectory analysis and stay points detection. Such Mobile Cloud Computing approach has been successfully employed for extending smartphone’s battery lifetime by exchanging computation costs, assuming that on-device stay points detection is prohibitive. In this article, we propose and validate the feasibility of having an alternative event-driven mechanism for stay points detection that is executed fully on-device, and that provides higher energy savings by avoiding communication costs. Our solution is encapsulated in a sensing middleware for Android smartphones, where a stream of GPS location updates is collected in the background, supporting duty cycling schemes, and incrementally analyzed following an event-driven paradigm for stay points detection. To evaluate the performance of the proposed middleware, real world experiments were conducted under different stress levels, validating its power efficiency when compared against a Mobile Cloud Computing oriented solution.

## 1. Introduction

Technological advances in the computation, sensing and communication dimensions of smartphone devices have contributed to their acceptance by users all around the world [1]. In particular, the smartphone’s sensing dimension is supported by a diversity of sensors that enables the unobtrusive acquisition of context information (e.g., location, user activity, ambient context variables, etc.) from raw sensory data [2]. Such context information represents highly relevant input for building personalized application services to enhance user experience, for instance, by adapting smartphone’s operation autonomously without requiring his or her intervention at all. Nevertheless, long-term *context-aware applications* must query sensors on a continuous basis, which could significantly impact on energy resources of smartphones [3]. This is because the battery capacity remains as the main challenge of mobile platforms, growing only 5% to 10% per year [4,5], or roughly doubling each 10 years [6]. Even worse, such growth rate is out of pace with respect to energy demands imposed by hardware components and the ever-increasing computing power offered by each new generation of mobile devices [7].

### 1.1. MCC as an Initial Solution for Addressing Smartphone Constraints

Early strategies for smartphone data collection and analysis adopted a Mobile Cloud Computing (MCC) oriented approach [2], where smartphone-based intensive computing, mass data processing, context extraction, and data storage are shifted to the cloud [8,9]. Such decision was made assuming that the limitations of computing and energy resources of mobile devices mark local processing as prohibitive. In the practice, MCC oriented solutions involve the transmission of raw sensory data streams (which could be quite dense) to the cloud and, after processing, send the response with the extracted information back to the mobile device. We argue that the fundamental reasons for avoiding local (herein after referred to as *on-device*) context extraction and processing in smartphone-based location sensing might not be valid for all the cases.In this matter, the continuous advances on computing and processing power of smartphones promote a re-evaluation of past assumptions about serious limitations of these devices to efficiently perform application specific processing tasks locally [10].

As an example of this rationale, the discovery of frequent places from smartphone collected raw GPS data has been usually addressed through an MCC oriented approach. The estimation of these frequently visited locations (herein after referred to as *stay points* or points of interest) of mobile users is a key enabler for large-scale and population-wise location and context-aware developments, such as city planning, transportation, and personalized communication systems [11,12]. Moreover, location information derived from stay points is highly related to moving and living patterns of individuals, and several studies have shown that the knowledge about user’s behavior and physical activities is a good indicator of human health status [12,13,14]. Thereby, the synergy of stay points detection and activity monitoring might provide medical experts with the ability to monitor and diagnose patients using continuously generated data, rather than data from a time-bounded medical appointment [14]. Such potentials motivate researchers to discover semantics from a stream of raw locations, which is an emerging trend in location based services (LBSs) [15].

**Figure 1:**
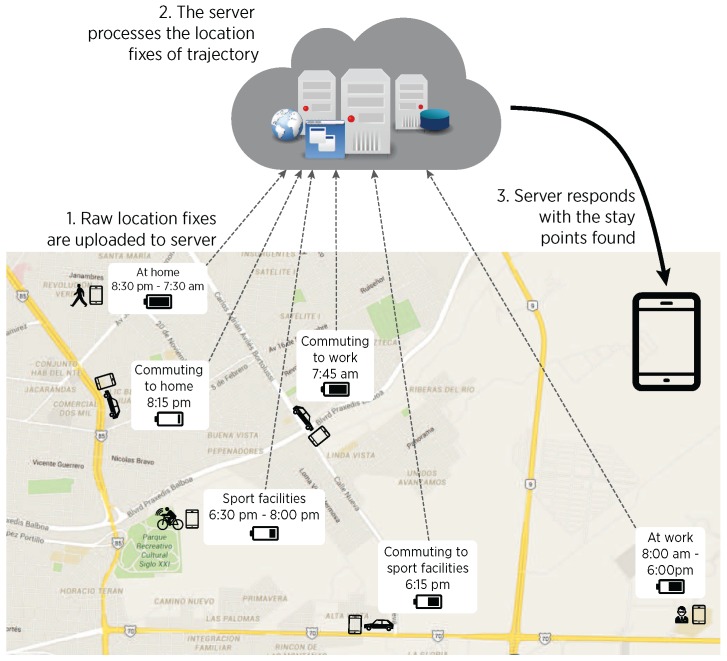
A typical MCC approach for stay points discovery.

In such LBSs, smartphones commonly obtain location information mostly via GPS receiver, as it offers the highest accuracy among the location providers available in modern smartphones [16] (e.g., Wi-Fi Positioning System and cellular cell tower location providers). Nevertheless, if fine spatial granularity is required by a given LBS, the GPS receiver must be queried continuously with a high sampling rate, which produces a high energy consumption because of the inherent characteristics of its infrastructure (synchronization and communication with GPS satellites [17]), as well as in an increased use of computational resources [18]. As a way to tackle this issue, existing solutions for stay points detection follow an MCC approach on which the fine-grained location fixes are optionally pre-processed locally, and then transmitted to dedicated servers for performing the analysis of full GPS trajectories, as conceptually depicted in Figure 1. Such offloading strategy has been normally preferred aiming to extend smartphone’s battery lifetime, as it has been assumed that mobile devices have limited computation resources [19].

### 1.2. On-Device Processing as an Alternative Solution

Although existing MCC oriented solutions are able to process full GPS trajectories (as in [12,20]), they still inherently perform such data analysis in an off-line manner, which represents a drawback when an on-line (live) detection of stay points is needed by applications with strict latency requirements, like vehicle and people tracking [21]. For these scenarios, it is impractical to wait until location data corresponding to a full trajectory is sampled, given that the immediate detection of relevant events would be discarded. Moreover, such issue is framed in a more global dilemma about the optimality of MCC oriented strategies for solving any mobile computing need [22]. Several guidelines for defining when to perform computation offloading have been proposed and studied [23,24], which raises the question of whether an MCC approach is optimal for any combination of computation, latency and tracking accuracy requirements. For instance, the transmission of location updates might not be possible due to security requirements, low battery level, or even simply because the mobile device is in a place without any wireless network coverage. Similarly, even if data transmission is possible, the time consumed while sending data over wireless interfaces, in addition to the time required for processing data at servers, could represent a critical issue for ensuring that the latency requirements of mobile applications are fulfilled.

An alternative solution that has been partially explored in the literature consists in the design of smartphone-targeted software platforms powered by algorithms that are aware of the impact of continuous computation and communication tasks in battery level, as well as aware of how to exploit the mechanisms for power and sensors management implemented by the mobile OS. The operation of such platforms could be driven by the event oriented nature of mobility changes, and could support the execution of location analysis techniques for full on-device and on-line detection of stay points.

Such alternative mechanism might also opportunistically label the identified stay points for adding semantic meaning using information from other sensors (e.g., accelerometer’s activity information, ambient sounds from microphone, etc.). Once computed, the semantic meaning and specific location and time attributes of stay points could be transmitted to the cloud, quite sparse instead of densely sampled raw location data, for large-scale or population-wise analytics. Hybrid solutions are also possible with consequences and tradeoffs in energy, accuracy, delays, etc. Moreover, far beyond of efficient on-device implementations, research is still needed for improving the power-efficiency of GPS position sensing using intelligent sensor management strategies [25]. We argue that following a full on-device strategy could improve the awareness of the mobile device and produce cognitive mechanisms that learn from user and enhance its operation autonomously. Additionally, since the amount of extracted information is smaller than the raw GPS data, the smartphone’s data transmission is likely to operate under a low-duty cycle regime, reducing energy consumption. Such data transmission savings could also reduce fees when mobile data networks are employed. Also, a full on-device implementation could reduce the dependency on cloud connectivity and help to prevent the security and privacy issues originated by MCC oriented solutions [26,27,28], since raw GPS fixes are kept on-device instead of being transmitted over the wireless media.

Attending the aforementioned motivation, in this article we propose and evaluate an event-driven and fully on-device stay points detection middleware, with support for duty cycled access to GPS receiver in a continuous manner. We adopted an event-driven design as it allows to interact transparently with the built-in location facilities of mobile devices. Similarly, it also acts in a power-aware manner as, out-of-the-box, it only activates its internal modules upon reception of a significant event notification, instead of performing a permanent data polling among its layers. Finally, the event-driven design eases the development and extension of the proposed middleware, as it favors a low coupling between its modules. The development of such middleware is not straightforward, as it must overcome several technical barriers imposed by the complex software stack of modern smartphones. For instance, although aggressive power saving mechanisms, changes in the power-states, and a too generic management of sensors aid the smartphone to obtain the most of its battery [29], they also contribute to hinder the implementation of power-aware continuous sensing. By addressing such barriers, our middleware is able to autonomously collect location updates in the background. Its event processing engine features an adapted version of a stay points detection algorithm [30,31] (although, as it will be described, any other differential algorithm could be employed), and it is prepared to process a stream of low-level events represented by the location updates, and transforming them into more complex events, corresponding to the detected stay points. The main contributions of this article are summarized as follows:

- The design of a full on-device middleware for autonomous location data collection and event-driven stay points detection in smartphones in a non-intrusive manner, which addresses power saving mechanisms of mobile OS and offers duty cycling support for a flexible sampling process.
- A quantitative evaluation through experiments in real mobile devices using the proposed middleware, which highlights the possibility of obtaining power savings when detecting stay points, in comparison with an MCC oriented solution, even under variable stress levels expressed as different fixed duty cycles.

From experimental results, we observe that only a minor degradation in the accuracy of detected stay points is produced, due to the employment of fixed duty cycles for accessing the GPS receiver. The middleware produces a minor computational impact that does not degrade the operation of the mobile device and, instead, reduces the energy consumption in comparison to GPS location transmission costs. The rest of this article is organized as follows. Section 2 summarizes background concepts and related work for identifying stay points from GPS data. Section 3 describes the proposed event-driven solution for on-device and on-line detection of stay points. The implementation of the proposed solution and the adaptations made for ensuring its execution in a smartphone are described in Section 4. Section 5 describes the experimentation performed and discusses the obtained results. Finally, Section 6 presents some concluding remarks and future work.

## 2. Related Work

In order to provide a self-contained description, we first present the definition of stay point, followed by a brief review of existing related work.

### 2.1. Stay Point Definition

A stay point refers to a geographical zone a user holds a special interest with. Formally, a stay point is a virtual location defined by latitude and longitude coordinates, as well as arrival time, and departure time. It is calculated from a set of time consecutive GPS fixes $P=\{p_{i},p_{i+1},\ldots ,p_{j}\}$, where $p_{i}$ is the first location fix of the trajectory, while $p_{j}$ the last one. Each GPS fix is in turn composed by latitude, longitude, as well as by the date time when it was collected, i.e., its timestamp (*ts*).

From the provided definition, stay points are constrained by a geographical region size and a stay time, so that in practice a distance $\theta_{d}$ and a time $\theta_{t}$ threshold parameters must be specified for their calculation. The GPS fixes of the set P must observe:

(1) $$\mathrm{distance}\left(p_{i},p_{x}\right)\leq \theta_{d},\forall \;i<x\leq j$$

(2) $$\left|p_{j}.ts-p_{i}.ts\right|\geq \theta_{t}$$

Equation (1) constraints the size of the stay point’s geographical area, indicating that the distance between the first location fix and any other contained in set P must be shorter than $\theta_{d}$. On the other hand, Equation (2) defines the minimum amount of time that user should spend inside a stay point, ensuring that the time interval between the timestamps (*ts*) of the first location fix ($p_{i}$) and the last one ($p_{j}$) is greater or equals to $\theta_{t}$. The centroid is considered as the representative location of a stay point, and its latitude (*lat*) and longitude (*lon*) coordinates are computed according to Equations (3) and (4), on which |P| represents the cardinality of P. The arrival time (*at*) and departure time (*dt*) are obtained from the timestamp (*ts*) of the $p_{i}$ and $p_{j}$ location fixes, respectively.

(3) $$\pi .lat=\frac{\sum_{k=i}^{j}p_{k}.lat}{\left|P\right|}$$

(4) $$\pi .lon=\frac{\sum_{k=i}^{j}p_{k}.lon}{\left|P\right|}$$

### 2.2. Stay Points Detection Strategies

Most of the existing MCC oriented solutions are powered by off-line algorithms on which the input is a full trajectory of time-sequenced GPS fixes and the output is the list of identified stay points [32]. In this sense, these algorithms could be categorized into three main strategies [33]:

- **Clustering-based strategies**: Clustering algorithms are employed for grouping raw coordinates into places. These algorithms could only consider spatial distance aspects (as in the DBSCAN algorithm [34]) or also involve the time dimension (as in DJ-Cluster [35] and CB-SMoT [36]).
- **Differential-based strategies**: Algorithms are based on the analysis of time and space differences between individual GPS fixes for finding centroids that represent a stay point (as in [30,31,37,38]). Because of the low computational complexity and streaming nature for calculating such differences, differential-based algorithms are more suitable for conducting on-line detection of stay points in smartphones [33].
- **Probabilistic-based strategies**: Algorithms under this category try to infer the most frequently visited places from location data by using probabilistic models, like Gaussian Mixture Models (as in [39]), Bayesian models (as in [40]), and Conditional Random fields (as in [41]).

Notice that since full trajectories are the input for most of these strategies, they are unable to incrementally compute stay points after each location update is received, which could enable on-line stay points detection. Similarly, their design often disregards the technical barriers for implementing them in mobile platforms, which are constrained by a limited battery, as well as by the availability and accuracy variations associated to GPS infrastructure. As a consequence, many of the existing MCC techniques only use the smartphone as a simple medium for collecting location data and, occasionally, for performing light filtering tasks.

### 2.3. Platforms for Location Data Analysis and Stay Points Detection

A rich body of research has been conducted for reducing the impact of MCC solutions on energy consumption due to the large amount of location data generated by the smartphone, and also for partially supporting on-device stay point detection. For instance, Barbeau et al., presented the *TRAC-IT* application [42], which implements the CP (Critical Point) algorithm and a location-aware state machine for reducing the amount of GPS data transmitted to server. The CP algorithm determines the relevance of a GPS fix, building straight trajectory lines where the fixes that do not contribute additional path information are omitted. Only critical GPS fixes are transmitted for further processing, reducing data traffic and power consumption. The location-aware state machine increments or decrements the GPS sampling period depending on the detection of movement by user or GPS coverage-availability. However, although TRAC-IT allows the energy-efficiently collection and transmission of GPS trajectories, the role of the smartphone is kept as a sensor with basic filtering operations, while the location data processing is performed on an external server.

ViBN, by Miluzzo et al. [12] is a continuous sensing mobile app for exploring multiple aspects of a city (like noise level) and its population, including stay points detection. ViBN employs a duty cycle scheme for accessing the GPS, following an MCC oriented approach that transmits a location fix only if user has been static. Upon smartphone’s request, a Density-Based Spatial Clustering of Applications with Noise (DBSCAN) clustering algorithm is executed on the cloud for detecting stay points, which are stored and transmitted to the smartphone for feedback purposes. Although the main reason for performing the detection of stay points at backend servers is the exploration of community sensing information, it could be realized that an on-device detection approach would further reduce the energy transmission costs and still allow population-wise analytics. Similarly, it would ensure an on-line detection and knowledge of stay points in the smartphone, instead of submitting refreshing requests.

Perez-Torres and Torres-Huitzil proposed in [20] a middleware for power-aware GPS data collection, which also offloads the computation of stay points. The design of the middleware supports the sampling of GPS receiver through a simple distance-based policy, which reduces the sampling interval (i.e., more frequent readings) if user moves more than a given distance threshold, or increments it when user is reported stationary. Despite the fact this middleware is able to extract stay points with the aid of cloud services, its main drawback is the off-line approach for performing their detection, which is also requested in an on-demand manner, as an optional feature instead of a continuous service.

Loseto et al., proposed a mobile agent [43] for profiling user behavior through a semantic scheme, which considers GPS and accelerometer data. Among its features is the on-line detection of stay points from GPS data in the mobile device. Authors refer to a slightly refined version of Montoliu’s algorithm [44] but such modification is not described. Although the purpose of this work is far beyond identification of stay points, its major drawback is the absence of the energy consumption as a system concern. For instance, the location data collection is not energy aware, neglecting power optimizations when employing location providers.

Lee et al., evaluated several algorithms for off-line trajectory analysis, and proposed an alternative method for stay points detection that employs a *superstate* model [33], as an extension to the classic Hidden Markov Models (HMM). Such method defines representative states built from mobility data, namely, staying indoors, staying outdoors, indoors, walking, and in transport. The user could be at any time in a *superstate*, which is modeled as the user being at all states simultaneously, each of them holding a likelihood; the state with the highest probability is selected as the current state. Across time, user mobility will describe transitions between superstates, which are hold in an HMM transition matrix. From these states, the system is able to identify stay points if, for instance, the special sequence of walking, indoors, staying indoors, and transportation states is detected. Nonetheless, the required learning stage is complex to achieve and its implementation in a mobile device was not demonstrated. Despite the quality of the results in stay points detection and behavior recognition of this work, the energy constraint was not considered in its overall design.

The on-line detection of stay points on-device has been partially addressed in consideration of its energy constraints. Hu et al. [38] presented a framework aimed at presence management for mobile instant messaging, which supports stay points detection. The framework is powered by an improved version of the algorithm proposed by Kang et al. [37], which supports noisy location updates and the quick returning events of user to a given location. It incrementally processes each acquired GPS fix for determining the existence of a stay point. Nonetheless, the system does not support GPS data collection using varying sampling periods or policies. Similarly, it lacks the support for changes in the active power mode of the smartphone, discarding further energy optimizations.

In this regard, existing work in the literature has been mostly focused in off-line detection of stay points, and the exploration of complementary schemes to the MCC oriented approach has been only partially analyzed. By studying such on-device alternative strategies, we consider that energy savings could be boosted (as densely data transmission is avoided), privacy concerns could be lessen (as data is kept in the mobile device) and autonomous cognitive features could be achieved by the smartphone itself.

## 3. Fundamentals of Event-Driven Solution

The overall design of the proposed middleware is described according to its two main features. The first one refers to the support for event-driven differential algorithms for stay points detection. The second one consists in an event-driven layered architecture that embeds the event-driven algorithms, autonomously manages the collection of location data (supporting duty cycling and policy-driven sampling), and notifies each identified stay point to LBS subscribers. Such design fits transparently with the way on which mobile platforms access to sensors. Both features are described in the following sections.

### 3.1. Event-Driven Adaptation of Stay Points Detection Algorithms

The asynchronous nature of GPS data acquisition promotes an event-driven design of the software stack of mobile platforms, or at least, as in the case of Android, a mock event-driven design that wraps the continuous polling between its layers [45,46]. Such design is even visible in the API (Application Programming Interface) for accessing location providers, which involves callback mechanisms for location updates notifications. However, most of the existing techniques for stay points detection are unaware of these mobile OS design aspects and, as a consequence, the event-driven design of mobility analysis services has been only partially exploited.

Among the different off-line trajectory analysis algorithms for stay points detection, the algorithm proposed by Li et al. [30,31] belongs to the differential-based family described in Section 2. As such, this algorithm exposes low computing requirements and, according to the evaluation performed in [33], it also achieves competitive accuracy results when compared against other algorithms. Because of these features, Li’s algorithm was selected and implemented in our work. Li’s algorithm steps are listed in Algorithm 1, and its behavior could be broadly described as follows:


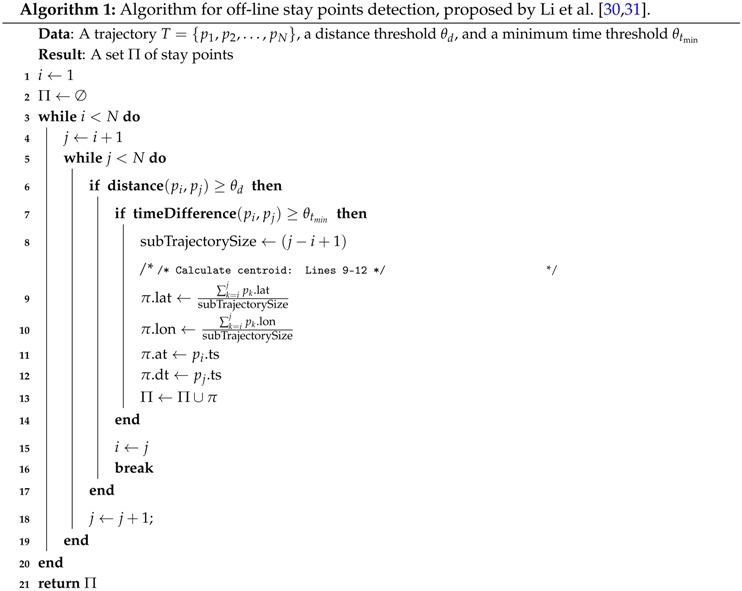

- First, the algorithm keeps two pointers $p_{i}$ (start) and $p_{j}$ (end) for defining a sub-trajectory that is iteratively accumulating more fixes, always within the distance and time parameter thresholds.
- Second, the $p_{j}$ (end) pointer is shifted to the right only when the distance constraint defined in Equation (1) is met, which is captured when the if comparison of line 6 is false, meaning that user is still inside the geographical region.
- Third, if the distance constraint is not met (i.e., the if comparison of line 6 is true), it would mean that user has exited from the geographical zone, so that the time constraint defined in Equation (2) (listed as the if comparison of line 7) must be evaluated.
- Lastly, a stay point will be built only when this last if comparison is evaluated as true, meaning that user stayed more than the $\theta_{t}$ time parameter inside the region defined by $p_{i}$, $p_{j}$ and intermediate location fixes. Regardless of the result of this last if evaluation, the $p_{i}$ (start) pointer is assigned the $p_{j}$ (end) pointer value in line 15, so that the analysis of sub-trajectory is restarted and the evaluation of the next location fixes is continued.

Li’s algorithm follows an inherent off-line approach for calculating stay points from a given trajectory. Nonetheless, as described in Section 2, differential-based algorithms are based on performing comparisons between individual location fixes, instead of analyzing clusters of them. As the source of location updates is an asynchronous stream of GPS fixes, such comparisons could be performed right after each location update is received. Following this guideline, Li’s algorithm, and any other off-line differential algorithm, could be adapted for observing an event-driven approach that allows the on-line detection of stay points. Once a new location fix that fulfils the algorithm’s specific conditions is received, a stay point could be generated and notified to subscribed modules.

Following the aforementioned guideline, we produced an adapted event-driven version of Li’s algorithm, shown in Algorithm 2, on which each arriving location update is analyzed for determining whether it should be inserted in the sub-trajectory being processed, instead of processing a full and runtime fixed GPS trajectory. The generic aspects of this adapted version relies on Lines 2 and 8, which define how each incoming event notification (i.e., the location update) is processed, and how the accumulated sub-trajectory data is reset, respectively. For instance, depending on algorithm design aspects, the Line 2 could represent the storing of the incoming location updates into an internal buffer, or just simply the accumulation of their coordinate values in the collectedData variable. As will be explained later in Section 4, such implementation variants are possible since differential algorithms rely on the centroid concept. Depending on how the location updates are collected and managed by the algorithm, Line 8 must properly reset the buffer or the accumulated location coordinates and then incorporate the $p_{\mathrm{last}}$ location fix.


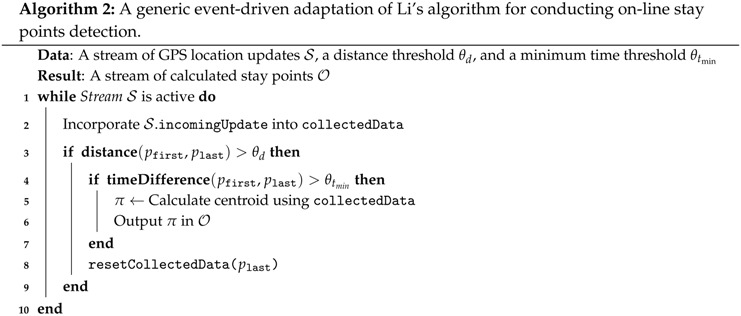


### 3.2. Mobile Software Stack Operation

Mobile platforms usually define a complex and rigid software stack as a way to overcome the constraints of their hardware components and to ensure the continuous execution of diverse computing, communication and sensing tasks. Such a design approach makes the power efficient collection of sensors data cumbersome, since a full understanding of the smartphone’s operation is needed. As a rule of thumb, it cannot be assumed that sensors data are immediately available upon request. In fact, there is an intrinsic asynchrony in the operation of sensors, since the collection of data requires to perform an actual measurement of the physical environment, situation that is augmented by the complexity of the software stack. Additionally, the measurement might depend on external infrastructure, as in the case of GPS location updates, which involves communicating with GPS satellites [17], introducing further delays in the acquisition of data. Thus, we preferred an asynchronous approach that defines an event-driven architecture for processing a stream of incoming GPS location updates.

**Figure 2:**
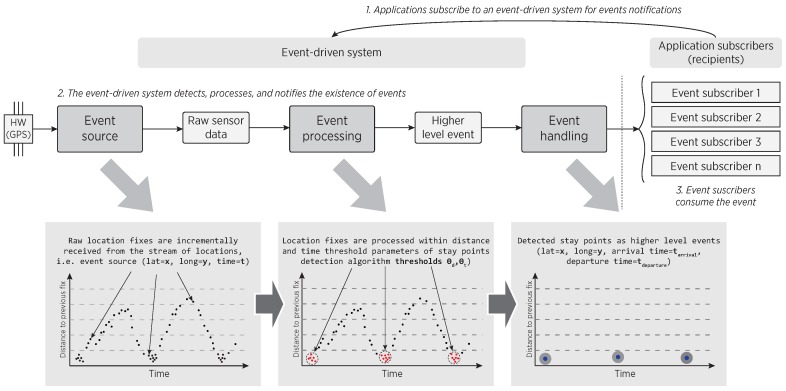
Top-level architecture of an event-driven system in the context of stay points detection.

An event-driven system is one that works by only sending asynchronous notifications (or message passing) between its components for triggering computing tasks [47,48]. Thanks to such a simple feature, the complexity of these systems is low, the coupling between their components is reduced and, since each component is only activated upon message reception, these systems are power-aware by design, theoretically reducing energy consumption when compared to typical synchronous systems [28,49,50]. The top-level architecture of an event-driven system consists of three logical layers: (i) *event source*; (ii) *event processing*; and (iii) *event handling* [48], as shown in Figure 2. The events have to be processed immediately so that a significant situation can be detected promptly.

Under the event-driven viewpoint, an *event source* is a system component that is capable of producing a stream of observations corresponding to events occurring in a given environment, for instance hardware sensors. As such, the GPS receiver is considered an event source, which continuously produces a stream of location update events. The next fundamental component is an *event processing* engine that incrementally processes the stream of incoming events and produces more complex events or actions with a valuable semantic meaning. In the proposed approach, the detection of stay points is kept in background, triggering processing tasks only when a GPS fix is received from the stream of location updates, and serving to concurrent applications, even during the smartphone’s stand-by power mode. More specifically, such processing considers the space-time relationship, transforming the simple low-level events (raw GPS coordinates) into stay points. Finally, an *event handling* component consumes the detected event and reacts accordingly by triggering a specific-domain action. The event-handling components must be registered in the driven system as event-subscribers in order to receive messages of incoming events. Such subscription-notification insight is usually supported by the mobile OS. For our middleware, the handling components refers to the subscribed mobile applications requesting stay points notifications.

Although not implemented in the presented work, the event handling or event processing could be improved to enrich stay points data using additional information, for instance: soliciting a semantic meaning of the location from user feedback (or from external servers); including activity information from accelerometer data; and performing further processing for detecting top stay points. These tasks could be also implemented by the smartphone itself following a personal sensing paradigm, having always the possibility to transmit this reduced amount of high level information to external servers for conducting population-wide analysis as in the community sensing paradigm [2].

## 4. Android Implementation

The proposed solution was implemented as a middleware for the Android platform, which provides fine-grained control for sensing operations and a flexible development environment. Nevertheless, the underlying principles of our middleware still are valid for other mobile platforms that also follow an asynchronous access to sensors. Specific actions were taken for ensuring the actual execution of the middleware in the smartphone, as described in next paragraphs.

### 4.1. Implementation of Event-Driven Algorithm

Taking the generic event-driven algorithm provided in Section 3 as a starting point, we derived and implemented two algorithm variants that differ on the way location data is accumulated and how stay points are calculated. Although the proposed solution makes use of the Li’s algorithm as a proof-of-concept, notice that its design is agnostic about the underlying differential-based algorithm. Therefore, alternative event-driven algorithms and strategies could be supported without requiring further modifications, as event-driven systems favor the loose coupling of their components [47,48].

#### 4.1.1. Buffered Event-Driven Algorithm

This variant uses an internal buffer for storing the location fixes of the current sub-trajectory. As such, the Line 2 in the generic Algorithm 2 aimed at incorporating the received location update is implemented by: (a) Assigning $p_{\mathrm{first}}$ the value of the first element of current sub-trajectory (i.e., subTrajectory.firstElement()); (b) Assigning $p_{\mathrm{last}}$ the value of the received location update; and (c) Adding the received location update to the sub-trajectory, which represents the collectedData variable (i.e., subTrajectory.add($p_{\mathrm{last}}$)).

The call to calculate centroid in Line 5 is a strict implementation of the stay point’s definition provided in Section 2. The length of the sub-trajectory is employed for calculating the average latitude and longitude coordinate values, marking the arrival and departure time of the stay point with the timestamp of first and last location fixes, respectively. Finally, the implementation of Line 8 for resetting data involves deleting all points of current sub-trajectory and then adding the $p_{\mathrm{last}}$ to it. In this way, the *buffered* algorithm only keeps in memory the minimum amount of location fixes needed for calculating stay points, instead of a full location trajectory.

#### 4.1.2. Sigma Event-Driven Algorithm

The event-driven *buffered* variant, as well as the original off-line Li’s algorithm, holds explicit memory requirements for storing the location fixes of current sub-trajectory. As such, it could be argued that a large amount of memory might be needed if user remains in the same place for a long time. A rough estimation of these memory requirements in the Android OS, considering a GPS sampling frequency of 1 fix per minute, is as follows. An instance of the native Location class (which represents a location fix) occupies 132 bytes, thus a full day of GPS readings could be allocated in around 185 kB. The memory provided by Android varies according to software version, mobile application and hardware constraints, and could be dynamically re-allocated when a mobile App reaches a low memory condition [51]. During several trials, 13.86 MB were initially allocated for our App, maintaining a free memory of no less than 2 MB, meaning that the buffering of location data might last more than 11 days. However, this situation is not likely since a user rarely describes such mobility pattern and the battery might be drained at that point. Despite assuming that the memory provided by Android is enough for a long time execution of *buffered* algorithm variant, we performed a slight modification, herein after referred to as *sigma*, which avoids the need for buffering GPS location fixes.

On this *sigma* version, the Line 2 for incorporating a location update is implemented by accumulating its latitude and longitude coordinate values, and incrementing the amount of accumulated fixes (i.e., the size of the sub-trajectory), as a way to perform an incremental calculation of stay points. The call to Line 5 is implemented as a simple arithmetical division of the accumulated coordinate values by the amount of fixes, attaching the timestamp of the first sub-trajectory fix and the received location update as the arrival and departure time of the stay point, respectively. Finally, the implementation of Line 8 resets the accumulated data by assigning the latitude and longitude coordinates of $p_{\mathrm{last}}$ fix, and sets the amount of sub-trajectory fixes to 1. In this way, the memory requirements for stay points detection are further reduced, ensuring the feasibility of its execution in the Android mobile platform.

### 4.2. Event-Driven Middleware for On-Device Stay Points Detection

**Figure 3:**
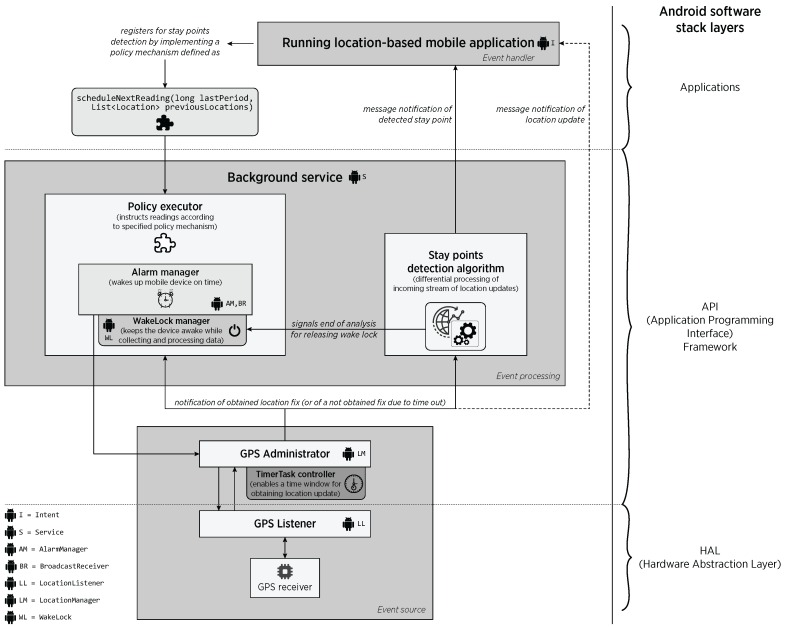
Architecture of the proposed middleware for GPS data collection and on-device stay points detection.

Figure 3 illustrates the architecture of the proposed middleware. We highlight the different Android API components used in our design, and particularly the deployment on the Android software stack. The modules of the architecture are activated upon the reception of message notifications from components of upper/lower layers. The event-driven design allow us to deploy a loose coupling architecture, implying that each module is agnostic about the processing details that originated the incoming message notification and instead, focus on processing the input event and producing the corresponding output that must also be notified as a message to other components.

Our middleware abstracts the complexity of the different layers and technical mechanisms that must be addressed for enabling continuous GPS access, like reacting to changes in power-level modes and energy management of Android OS. Its architecture provides significant enhancements of our previous work (described in [20]), improving the way on which mobile LBSs register within the middleware, adding support for flexible definition of policies through Dependency Injection [52], proper adaptations due to changes in Android platform, and most importantly, the possibility of performing the stay points detection entirely on the mobile device. Since the event-driven approach is based on message passing, we employed the Observer pattern throughout our middleware to register subscribers for receiving notification about changes in the platform [53], specifically the reception of location updates and the detection of stay points.

A detailed description of how the middleware employs such Android low-level components and the interaction between its modules is as follows. Initially, at the top of the Android software stack (i.e., the Applications layer), the *Running location-based mobile application* requests the detection of stay points to the *Background service* component, using an implementation of a Java interface that describes the policy to follow for subsequent GPS accesses. As shown in Figure 3, the interface defines the method scheduleNextReading(long lastPeriod, List<Location> previousLocations), where the lastPeriod parameter refers to the last time interval employed for scheduling GPS readings, while previousLocations contains a list of the last location fixes reported by the GPS receiver. The interface implementation is evaluated by the *Policy executor* once a new location update is received, allowing the middleware to define the moment on which next reading must be scheduled.

By using the interface, the hosted mobile app is able to inject this functionality according to its own needs. For instance, it could study the distance and time changes of last obtained locations, generating speed-based policies (as in [54]), or simply return a fixed value, so that a static sampling period would be employed for accessing GPS. Technically, the request for stay points notifications is performed by an Activity of the hosted mobile app, which establishes a connection with a Service (the *Background service* component) using an Intent when calling the bindService() method. After such method call, the *Background service* is started in foreground mode, marking it with high priority so that Android OS does not kill it under low memory scenarios.

The *Background service* component includes several modules built from different Android API Framework elements, in particular the alarm, power, and location system services. After the *Policy executor* evaluates the interface implementation, the next GPS reading is scheduled by registering alarms into the mobile OS using the AlarmManager system service of the Android software stack. Among the different task scheduling strategies provided by Android, the *AlarmManager* approach is the only control point for addressing the aggressive power-saving mechanisms aimed at extending battery life. In this way, the smartphone could be on its active mode, even reactivating it from sleep mode (also known as *doze*) if needed. The scheduling of alarms is followed by the registration of a BroadcastReceiver component that will receive event notifications of alarms going off, so that the middleware is reactivated for continuing location data collection.

Although scheduled alarms are guaranteed to be triggered, the smartphone still can return to sleep mode while collecting and processing GPS data. The Android OS’s POWER_SERVICE system service provides a specific token denominated partial WakeLock for explicitly controlling the power state of the mobile device. When acquired, a WakeLock forces the CPU and low-level hardware components to be kept in active power mode. We employed a specific type of WakeLock denominated PartialWakeLock, as it is able to turn off the screen while keeping the CPU and sensors awake. In this way, the *WakeLock manager* component ensures that the smartphone will be kept awake when accessing to GPS and processing location data [55].

The *Background service* also embeds the *Stay points detection algorithm* module, which executes the event-driven *buffered* and *sigma* algorithm variants for on-line detection of stay points. This module is aware of the limitations of smartphone’s battery, so that after notifying the detected stay point to the subscribed mobile app, it also notifies to the *Policy executor* the end of location processing for the immediate release of the active WakeLock. The proper management of WakeLocks is essential for the continuous operation of mobile apps, and their unexpected retention and missuses are common causes of a poor management of energy resources [55]. Another critical aspect, described later on, is that location updates might not be obtained under specific circumstances. Here, it is important to state that the event-driven variants of Li’s algorithm employed as proof-of-concept are able to deal with intermittent acquisition of location updates. More specifically, these algorithms are able to generate a stay point even if only the initial and last location fixes at the entrance of a stay point are acquired. Nevertheless, other algorithms and further analysis processes are encouraged to handle the issue.

The GPS administrator and listener layers control the registration - unregistration with the GPS hardware. The *GPS Administrator* module employs the LocationManager Android API component for accessing to the native GPS location provider, using the sampling scheme instructed by the *Policy executor*. The *GPS Listener* module, upon *GPS Administrator* request, registers for location updates with the Android HAL (Hardware Abstraction Layer) using a LocationListener implementation. Upon location update reception, the *GPS Listener* unregisters from HAL so that GPS receiver could be turned off, and notifies the location update to subscribers in upper layers of the middleware. It is important to recall that the acquisition of a GPS location fix is highly dependent on the visibility of GPS satellite infrastructure, which is affected in indoors locations, and by other external factors like meteorological phenomena. As a consequence, it is possible that location updates could not be retrieved. For this issue, the *GPS Administrator* includes a TimerTask mechanism that provides a time interval on which the location update must be obtained. In the case a time out is reached, the *GPS Listener* is unregistered from the native location provider and a notification of *not found location* is delivered to upper layers. The subscribers of location updates, i.e. the *Policy executor* and the *Stay points detection algorithm* modules, must be aware of this issue and prepared to react accordingly.

Despite the fact Android offers a mock event-driven API for sensors access, its actual implementation instructs layers to pull data from underneath layers [46,56]. Unlike this, the proposed middleware is powered up by an event-driven approach, on which each of its layers pushes data to above layers by using message notifications. In addition, it includes the mechanism for trying to obtain a location update within a strict time interval, which avoids a prolonged use of GPS when the connection to satellites is unlikely to be successful. These characteristics provide an improved management of sensors, computing, and energy resources of the mobile device, which aims to fill the lack of continuous sensing support exposed by Android.

**Figure 4:**
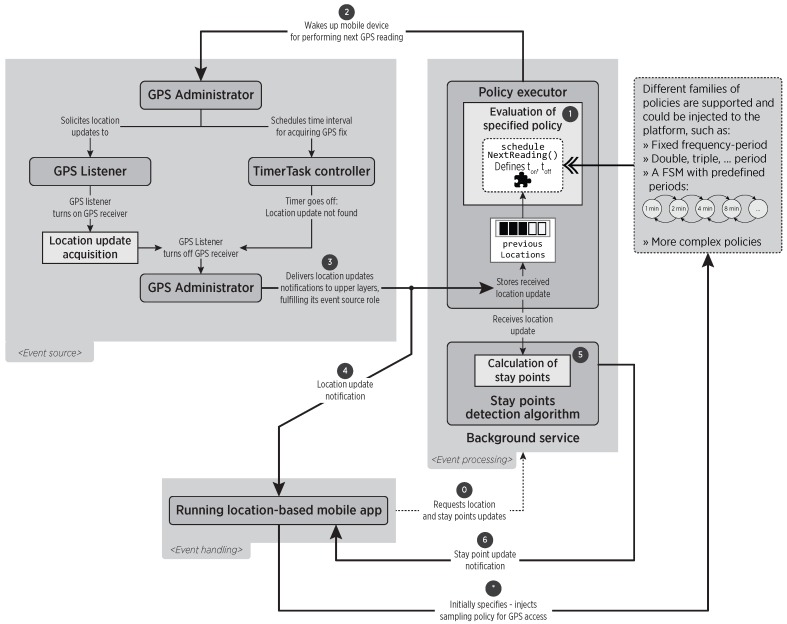
Interaction between components and information flow across tasks executions. Components are provided by the middleware itself, with the exception of the policy (shown as dotted) that could be injected by an additional entity, such as the hosted mobile app.

Figure 4 shows a pragmatic vision of the tasks performed by the middleware upon request of location and stay points updates. After specifying the sampling policy to use (in the step denoted as *), the *Running location-based mobile app* requests the notification of location and stay point updates to the *Background service* in step 0. Then, the *Policy executor* evaluates the specified policy (Step 1) for scheduling the time at which the mobile device must be woken up (Step 2) to perform location updates. After the location update (or a time out) is obtained from the event source components (*GPS Administrator* and *GPS Listener*), it is notified to upper layers (Step 3), including the hosted mobile App (Step 4). The location update is received by the *Background service*, for launching the event processing in the *Stay points detection algorithm* module (Step 5), which would notify the detection of a stay point to the hosted mobile App (Step 6). The location update is also processed by the *Policy executor*, which after storing it inside the previousLocations list, will trigger the *Evaluation of the specified policy*, restarting the sampling process.

Thus, the *Policy executor* block could analyze a historical window of GPS data to infer context information about user mobility and generate an adequate schedule for the next GPS access. Although in this article the analysis of the previousLocations list is discarded and only fixed sampling periods are explored, such scheduling could be based on more complex mechanisms that employ information learned from multi-sources of context data [2,29]. It is important to remark that the processing of any data collected from smartphone’s sensors, especially from location providers, is affected by security and privacy concerns of users in regard to exposing personal information. Although we recognize this issue, we focus on the technical and power aspects of the challenges imposed by power efficient stay points detection in mobile platforms.

## 5. Experimental Results and Evaluation

Experimental trials were performed in order to evaluate two main aspects of the proposed middleware. On the one hand, we evaluated the feasibility and reliability of employing it for conducting stay points detection completely on-device. On the other hand, we analyzed the energy savings produced by its event-driven design when compared against an MCC approach that transmits location data for offloading the detection of stay points.

For all of the outdoor experiments, the smartphones employed were the Google Nexus 6, which are equipped with a 2.7 GHz quad-core processor, 3 GB RAM, and a new 3220 mAh battery, running Android 6. As part of fair experimental conditions, the smartphones were only employed for running the experimentation, discarding any other uses (like phone calls and messaging), no additional mobile apps were installed on them, and only the mobile app corresponding to the experiments was kept in foreground, using the GPS as the unique location provider (wireless networks and cellular location providers were disabled). The parameters values for running the stay point detection algorithms were set to 45 min for $\theta_{tmin}$ and 500 m for $\theta_{d}$. Additionally, a Qstarz BT-Q1000eX GPS logger unit was also carried, acting as a ground-truth GPS data collector with a sampling frequency of 1 Hz, for establishing a baseline to study the performance of stay points detection for different GPS sampling intervals. During all of the tests, the GPS logger and the two smartphones were permanently carried together, so that the network connectivity status and GPS signal reception could be kept as similar as possible for all of the devices. With the aim of obtaining consistent stay points, the trials were conducted ensuring that user followed the same trajectories and visited the same places under similar schedules. Specific measures taken for individual experiments are described in the following sections.

### 5.1. Feasibility of On-Device Stay Points Detection

The main goal of the first experiment was to ensure the feasibility of performing on-device stay points detection using the proposed middleware. In this regard, the feasibility is understood as the ability for executing the middleware in the mobile device under different stress levels without a premature finalization caused by CPU and memory consumption usage issues. For this purpose, the experiment consisted on developing a sample Android location-based mobile application that would request stay points detection to our middleware. The referred stress levels were implemented by the sampling periods in the set {30, 60, 90, 120, 150 s}, which produce different workloads in the CPU, memory, and battery consumption of the smartphone.

The selected sampling periods defined fixed duty cycling schemes for the GPS receiver that, although simple, are still valid for the purpose of the experiment. Note that in this article we do not focus on a deep quantitative evaluation of the correctness of duty cycling schemes towards accurate location tracking and stay points detection (as performed in [54]). Instead, we focus on ensuring that the event-driven design of the proposed middleware could support duty cycling (as a feature) for collecting location data while properly handling the produced CPU and memory workloads.

The experimentation was performed assuming that typical speeds from transportation modes are [57] (a) stopped 0–1 m/s; (b) walking 1–2 m/s; (c) biking 2–5 m/s; and (d) motor 5–20 m/s, so that the aforementioned sampling periods could keep a spatial granularity in the range of 30–3000 m. For each round of the experiment, a campus student carried the GPS logger and two Nexus 6 smartphones with a fully charged battery, with one phone running the *buffered* algorithm variant, and the other mobile device running the *sigma* variant, both of them using the same sampling period at once.

The results of the first experiment are summarized in Table 1. The column *sampling period* indicates the sampling period employed for accessing the GPS receiver, while the *algorithm variant* column indicates the version of the algorithm executed by the mobile device. Column *obtained GPS fixes* refers to the amount of GPS fixes that were requested by the middleware to the mobile OS and that were actually obtained. It is important to mention that a 1 min timeout was established for obtaining each location fix; if the timeout was reached, the GPS location fix was considered not valid and a new GPS reading was scheduled. The column *average GPS fixes per SP* indicates the average amount of location fixes involved in all of the calculated stay points. Such amount gives an idea of the amount of memory that the smartphone should manage for executing the *buffered* algorithm variant. Finally, the *running time* indicates how long (min) the experiment was executed by the smartphones, starting from a full-charged battery and ending with a 4% battery level (such battery level was employed so that the last stay point could be calculated before the smartphone was shut down due to battery depletion).

For all of the sampling periods, both smartphones were able to calculate stay points without being affected by their memory and computing capabilities, ensuring its feasibility. By analyzing the performance of each algorithm during the trials, it is noteworthy that the usage of larger sampling periods incremented the total running time of each trial, with the exception of the outlier represented by the *buffered* version with the *60 s* sampling period, which describes a running time even higher than the obtained in the *150 s* sampling trial. Such outlier could be caused by the multiple background tasks that smartphones perform and that affect the transitions to the different internal power states of the mobile platform. Similarly, as could be intuitively inferred, shorter sampling periods could obtain higher amounts of GPS fixes in each algorithm variant, again with the same outlier of *60 s* sampling trial. Also, it is noteworthy that the *running time* does not exactly correspond to *obtained GPS fixes* × *sampling period* value since each fix is not obtained immediately and because of the aggressive internal mechanisms that the Android OS implements for reducing energy consumption in the smartphone’s idle state like the *doze* and *app standby* features [58].

In this regard, any attempt for replicating the presented results must consider such encapsulated power saving mechanisms that do not expose control points to developers and that hinder the execution of continuous GPS sampling. The effect of the power saving mechanisms is evident in the experiment’s results, since for all of the tested sampling periods one algorithm implementation depleted the battery faster than the other one, describing a shorter *running time* even though both smartphones were kept under the same described environment. Additionally, an extra energy overhead is generated when a GPS fix is requested by the mobile app to the mobile OS, but it is not obtained due to severely attenuated signals. Such weak satellite signals are highly dependent to factors like the location of user (e.g., indoors) or mobility conditions that, although identical for both smartphones, are out of control in the experimentation’s scenario. As a result of the previous factors, there is no a unique algorithm variant that improves the running time for all of the sampling periods of the conducted experimentation. As such, despite the fact that memory requirements of the *buffered* algorithm version are higher than those of the *sigma* variant, it was not possible to elaborate conclusive remarks from the conducted trials about the energy performance of the algorithms. More experiments are needed for detecting a confident tendency in the energy performance improvements of one algorithm variant respect to another. Nonetheless, it is important to recall that the main objective of this experiment was to ensure the feasibility of employing the proposed middleware towards stay points detection, which was successfully proved.

#### Accuracy of Stay Points Detected through Different Sampling Periods

In addition to exploring the feasibility of performing on-device stay points detection, the first experiment also allows to evaluate the impact of non-continuous GPS sampling on the spatial and temporal accuracy of detected stay points. In this matter, it is important to consider that there is no restriction in the density of the GPS trajectories employed for detecting stay points, so that stay points calculated from non-continuous trajectories could be compared against stay points generated from ground-truth (i.e., continuous) trajectories as follows. Let $x=\{x_{1},x_{2},x_{3},\ldots ,x_{i}\}$ be a stay point composed of *i* fixes collected from ground-truth data, and let $\tilde{x}=\left\{\tilde{x_{1}},\tilde{x_{2}},\tilde{x_{3}},\ldots ,\tilde{x_{j}}\right\}$ be a stay point composed of *j* fixes collected through a specific sampling policy, and also let i>j (i.e., the ground-truth stay point was calculated using more location fixes than the sampling policy stay point). The characteristics of accuracy in the time dimension can be expressed as follows:

(5) $$\mathrm{Arrival}\;\mathrm{time}\;\mathrm{difference}\to |x_{1}.ts-\tilde{x_{1}}.ts|<\varepsilon_{at}$$

(6) $$\mathrm{Departure}\;\mathrm{time}\;\mathrm{difference}\to |x_{i}.ts-\tilde{x_{j}}.ts|<\varepsilon_{dt}$$

(7) $$\mathrm{Stay}\;\mathrm{time}\;\mathrm{difference}\to |\left(x_{i}.ts-x_{1}.ts\right)-\left(\tilde{x_{j}}.ts-\tilde{x_{1}}.ts\right)|<\varepsilon_{st}$$

On the other hand, the spatial accuracy is evaluated by a direct measurement of the distance between stay points.

Table 2 summarizes the time and spatial differences between stay points calculated by smartphones against those calculated from ground-truth location data (GPS logger device), using the aforementioned strategy. The column *detected SP’s* indicates the amount of stay points obtained by the algorithm variants in each experiment round, while the *average SP stay time difference* and *average SP distance difference* columns show the time and spatial differences from ground-truth location data. As it could be expected, the shortest sampling periods calculated stay points more accurately, in terms of distance to the ground-truth stay points, when compared against largest sampling periods.

Note that for some of the experiment rounds, the algorithm variants detected less stay points than those according to ground-truth values, which was due to the depletion of battery that caused the finalization of the mobile app. The fact that battery ran out faster for one smartphone is, again, attributable to several factors like the different tasks that mobile platforms run in the background (which represent an energy overhead), and the power saving mechanisms implemented by the mobile OS. In any case, regardless of a lower running time, the semantic meaning of the stay points detected by both smartphones was equivalent. There was only one actual missed stay point that occurred when executing the *sigma* algorithm variant with a *150 s* sampling period, caused by accuracy issues of the smartphone’s GPS receiver. It is important to highlight that the accuracy results shown here are highly dependent on the actual mobility of smartphone user, which is one of the common issues that hinders the comparison of power-aware frameworks for LBS [29].

**Figure 5:**
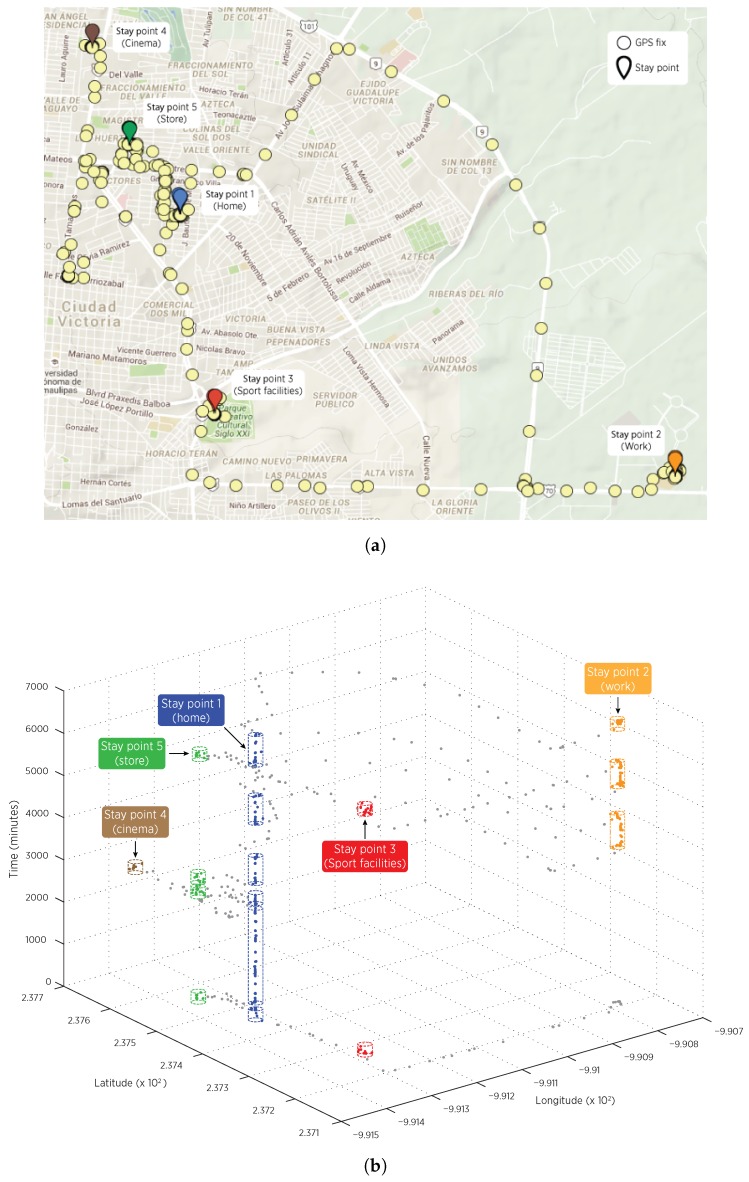
Location fixes and stay points rendered as pins in maps. The temporal aspect is discarded. (**a**) Location fixes and stay points rendered as grouped cylindrical regions in a three-dimensional coordinate system composed by longitude, latitude and time; (**b**) Visualization of a set of stay points found in the experimentation.

We also validated the correctness of the obtained stay points by analyzing the graphical representation of part of the outcomes from the first experiment. In particular, we considered a sub-trajectory obtained using a *30 s* sampling period that denotes the mobility pattern of a user that leaves home in the morning, stays around 9 h at work, and then arrives to a sport facility for returning home later. On such sub-trajectory, different motion speeds (in the range of a vehicle transportation mode) were described by user, which produced stay points with different spatial granularity. The Figure 5a shows the obtained stay points in a typical map visualization, discarding temporal features. On the other hand, the Figure 5b adds the time dimension for showing stay points as three-dimensional regions delimited by cylinders. For any cylinder, its radio is in function of the minimum distance $\theta_{d}$ parameter value employed by the stay points detection algorithm, while its height is at least the value of the minimum time threshold $\theta_{t_{min}}$ parameter. Such three-dimensional representation provides an additional perspective for understanding stay points as clouds of GPS locations close in both time and spatial dimensions. A total of 5 different stay points were effectively detected in the considered sub-trajectory. Notice that such temporal visualization in Figure 5b enriches the analysis as it shows the number of occurrences of a given stay point. In particular, home location corresponds to the stay point # 1, which was visited 6 times within the trajectory duration.

From these results, we also identified the possibility of performing an on-line and on-device stay points detection using location data collected through duty cycling schemes that, in conjunction with the event-driven design of the proposed solution, could reduce energy consumption without producing a significant impact on the spatial and time accuracy of calculated stay points.

### 5.2. Energy Savings of On-Device Stay Points Detection

The objective of the second experiment was to bring the smartphone under different stress levels for evaluating the energy consumption of the proposed middleware with respect to an MCC oriented solution that offloads location data processing. For this experiment, in the first smartphone we implemented the on-device middleware architecture, whereas in the second one we deployed a location application based on an MCC approach.

The stress levels were the same sampling periods in the set {30, 60, 90, 120, 150 s}. The smartphone running the mobile app hosted by our middleware executed the event-driven *sigma* algorithm for performing the on-device discovery of stay points. In the case of the MCC oriented solution, it also subscribed for location updates to our middleware, but translated each received location fix into a string representation (consisting on 116 bytes, in average) for its immediate transmission to an application server using the available cellular data network. As in the previous experiment, both smartphones were carried always together, following the same trajectory. Regarding wireless interfaces, only the smartphone running the MCC oriented mobile application was allowed to access mobile data network. Also, both smartphones had the Wi-Fi interface enabled, but were not allowed to connect to any access point during the trials.

The results of the experiment are summarized in Table 3. The GPS-on time column refers to the estimated effective time (min) that the GPS receiver was turned on for acquiring location fixes. The column *average acquisition time per fix* indicates the average amount of seconds invested for acquiring each location fix, which is calculated as the quotient between GPS-on time and the amount of obtained GPS fixes. Finally, the *data sent* and *data received* columns, applicable only to the MCC oriented tests, include the total amount of bytes requested by the mobile app to be sent as payload and the total amount of bytes received as payload in the smartphone by the server side, respectively.

From these results, it could be observed that all of the local executions, regardless of the employed duty cycle, outperform the running time of the corresponding remote trials, increasing the battery lifetime in factors within the range 1.26**x** to 1.52**x**. This produces a more efficient usage of energy resources and allows to conduct location tracking and stay points detection for a longer time. On the other hand, the MCC oriented strategies achieve shorter running times and obtain a lower amount of GPS fixes than corresponding local trials, which is caused by the energy overhead generated by the data transmission that contributes to a faster depletion of battery. It is also worth to note how the average time for obtaining a fix in the local trials is increased when longer sampling periods are employed, which could be explained by the different types of power states in the operation of GPS receiver (cold-start, hot-start, and warm-start). Longer sampling periods might represent a heavier energy demand since GPS receiver should perform a cold-start for obtaining a GPS fix. The average acquisition time of GPS fixes in the MCC oriented implementations is lower than the on-device strategy, which is attributable to the assistance provided by the mobile data network to the A-GPS receiver of the smartphone [17,59]. However, energy savings are still offered by on-device stay point detection when increasing the underlying sampling interval.

**Figure 6:**
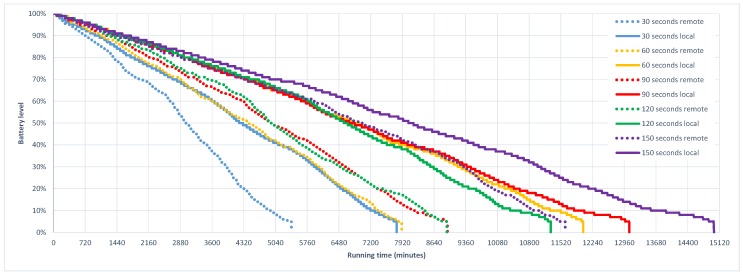
Energy performance comparison of on-device vs MCC oriented sample apps using different GPS sampling periods.

Figure 6 shows the energy consumption performance across time of both strategies for all of the employed sampling periods. The results shown on the plot were mapped to a sampling-independent timing format where the elapsed time from the beginning of the experiment was considered for evaluation and representation. As it could be expected, shorter GPS sampling periods have a higher impact in battery performance, which reduces its level quicker than longer sampling periods. Note also that for all cases the MCC oriented solutions produce a higher battery consumption than their on-device equivalent. In particular, it could be observed from the plot how the different tests corresponding to remote versions are finished in almost a sequential order (i.e., trials end in ascending order of its sampling period) with the exception of the *120 s* test, which ends very close to the *90 s* trial, but 15 min before. In the case of the local tests, a similar situation is found, where tests end following an increasing order of their sampling period, again with the exception of the *120 s* sampling trial that finishes 739 min before the *60 s* trial. The unexpected early finalization of the *120 s* trials, both in remote and local tests, suggests that this specific sampling period could represent a breaking point in the operation of the GPS receiver of the employed smartphones. A pragmatic interpretation is that location update requests in a *120 s* sampling period produce cold and/or warm starts in the operation of GPS receiver. This means that for some of the sampling periods, the ephemeris and almanac information of GPS satellites must be updated, producing an additional energy overhead that increases energy consumption, up to the point of finishing the execution of the trial even before than shorter sampling periods.

From the energy performance results, it is possible to explore the battery gains obtained by the on-device implementation. Table 4 summarizes the energy savings observed during the experimentation at different instants, expressed as percentages. Such percentages are normalized, as the different experiments achieved different running times. More specifically, 0% percent indicates the beginning of both tests, while 100% is the running time of the remote test (that is, the largest elapsed time at which both trials were simultaneously executed). As an example of these time differences, the actual elapsed time for a 20% of the *30 s* sampling period experiment corresponds to 1080 min; while for the *60 s* sampling period such percentage is equivalent to 1581 min. The battery gains shown in Table 4 are calculated according to Equation (8), where both battery levels correspond to the same time interval.

(8) $$BatteryGains_{t_{i}}=\frac{LocalBatteryLevel_{t_{i}}-RemoteBatteryLevel_{t_{i}}}{RemoteBatteryLevel_{t_{i}}}$$

An example of the battery gains calculations, in this case for the 80% elapsed time of the *30 s* sampling period experiment, is as follows. 80% of elapsed time in the remote experiment corresponds to approximately 4321 min; at that time, the level of the smartphone’s battery running the on-device implementation is about 49% and for the MCC oriented strategy is around 20%. From Equation (8), it is calculated as

$$BatteryGains_{80\%}=\frac{49-20}{20}=1.45$$

**Figure 7:**
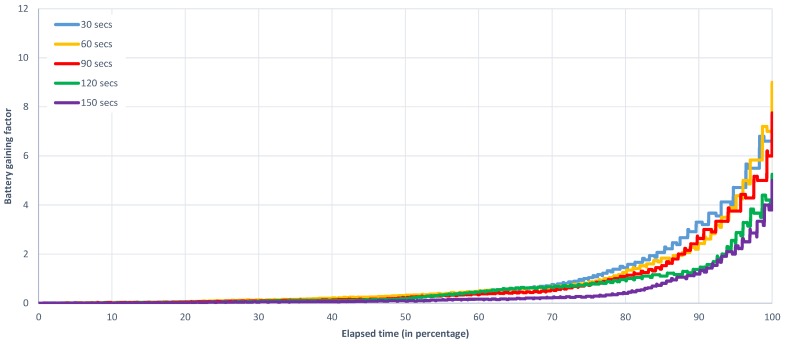
Battery gains obtained by the proposed on-device approach in the different experiments.

A practical interpretation of the obtained gain values is as follows. Values close to 0 represent no improvements in battery performance, and hence it can be concluded that both, on-device and MCC implementations, produce the same energy consumption. On the other hand, a value close to 1 indicates that the on-device implementation is doubling the energy performance of the MCC strategy. Figure 7 shows the evolution across time of the battery gains achieved by the proposed on-device stay point detection strategy. The x-axis denotes the normalized total elapsed time of each experiment trial until the battery of the smartphone running the MCC oriented implementation is drained. In this regard, it is noteworthy the non-linear performance of batteries when reaching the empty level, as their discharging rate is accelerated. This can be identified as the battery gains are incremented when the battery is near depletion in the smartphone running the MCC oriented solution.

From the results of the second experiment, we find a tendency recalling the power benefits of the proposed on-device middleware. However, these results can not be generalized to other scenarios considering different mobility patterns. In order to have further insight into energy gain factors, more experimental trials are needed so that a better explanation of the battery depletion in function of the different sampling periods, mobility of user, and underlying technical aspects could be given. It is noteworthy the existence of issues related to the accuracy of GPS fixes obtained by the smartphone. Because of these issues, some stay points were prematurely calculated, as the reported GPS fixes described changes in the location that were considered as the user leaving a stay point. Nonetheless, as the experimentation’s results show, there is room for producing power savings by means of a policy manager that could adapt the duty cycle of the GPS receiver and at the same time, obtain an accurate location tracking of user, as is shown in [60,61,62]. Such adaptive duty cycling scheme could be based on context data coming from different sensors as well as from information extracted by machine learning techniques that would allow to make predictions about user mobility. For instance, it could be possible to adapt the duty cycle of GPS to achieve a power-aware stay points detection, as well as using the discovered information for defining power-aware policies that improve the energy efficiency when accessing to location providers. In any case, providing the smartphone with the ability of calculating stay points locally allows to increase its awareness about mobility patterns of user. Thus, the proposed event-driven mechanism could represent a starting point for enabling cognitive features in the mobile device, in such a way that, by itself, it could learn and decide when to reinforce the mobility information discovered through its event processing layers.

## 6. Conclusions and Future Work

This paper has presented an event-driven middleware strategy aimed at on-device stay points detection from live GPS location data. The experimentation conducted shows that the proposed solution can be executed free of computing and memory issues in modern smartphones and potentially allows to reach always-on context awareness. Although verified through fixed GPS sampling, the design of its architecture is yet valid for following adaptive sampling strategies for collecting GPS trajectories. From the experimental results, it is identified a tendency for reducing the energy consumption when compared against an offloading MCC oriented approach, suggesting that computation is much more optimized (cheaper in power consumption terms) than wireless transmission in the tested mobile platforms and under the discussed scenario. Since the proposed on-device approach avoids the high energy consumption generated by the wireless transmission of densely sampled locations, it is able to calculate stay points with a good precision and without jeopardizing energy resources of the mobile device. Moreover, it was outlined that collecting and analyzing stay points and mobility patterns completely on-device provide an opportunity for optimizing diverse system design requirements, such as the energy consumption that could be saved when reducing the data traffic, and the performance of location-based mobile apps that could infer and learn the stay points without delays caused by computation offloading.

As future work, the fluctuations in the accuracy of GPS data collected by smartphones can be addressed through preprocessing techniques that smooth such inherent error in subsequent location updates, or by including data from additional context information sources, like inertial sensors and wireless communication interfaces. Similarly, the exploration of more alternatives for full on-device discovery of stay points is needed, which could be achieved by adapting more classic trajectory analysis algorithms to an event-driven perspective. As previously mentioned, the *Policy executor* component of our middleware could be improved by supporting policies that involve multiple sources of context information as well as machine learning techniques that provide a characterization of the places where an individual spends most of the time, building an expanded spatial-time model of user mobility. Such a mobility model could be exploited for improving the management of GPS receiver and produce an adaptive duty cycling that performs accurate location tracking with power efficiency. Aligned with this insight, it is important to remark the current trends that visualize the smartphone as a device capable, on its own, of performing context information discovery through adapted machine learning and pattern recognition techniques. Such perspective increases the variety of application niches and the substantial benefits for people on their daily activities.

## Figures and Tables

**Table 1:** Summary of results of first experiment (SP = stay point).

Sampling Period (s)	Event-Driven Algorithm	Obtained GPS Fixes	Average GPS Fixes per SP	Running Time (min)
30	Buffered	3876	218.3	6752
	Sigma	5199	244.4	8243
60	Buffered	5307	155.6	14,877
	Sigma	3054	126.3	8428
90	Buffered	2573	115.2	7694
	Sigma	2447	108.1	7522
120	Buffered	1708	77.4	8460
	Sigma	1993	82.2	10,214
150	Buffered	1417	53.8	10,433
	Sigma	1651	51.1	10,349

**Table 2:** Spatial and time accuracy observed in results of first experiment (SP = stay point).

Sampling Period (s)	Event-Driven Algorithm	Detected SP’s	Average SP Stay Time Difference (s)	Average SP Distance Difference (m)
30	Buffered	16 (out of 19) ^†^	64.13	13.35
	Sigma	19 (out of 19)	68.78	16.01
60	Buffered	29 (out of 29)	98.24	14.97
	Sigma	21 (out of 29) ^†^	82.35	19.42
90	Buffered	20 (out of 20)	104.95	20.6
	Sigma	20 (out of 20)	211.68	20.68
120	Buffered	19 (out of 21) ^†^	63.7	35.56
	Sigma	21 (out of 21)	59.7	34.05
150	Buffered	24 (out of 29) ^†^	116.4	59.11
	Sigma	28 (out of 29) ^‡^	115.6	50.81

^†^ Due to battery depletion; ^‡^ Actual SP miss.

**Table 3:** Summary of results of second experiment.

SamplingPeriod (s)	Processing Strategy	Obtained GPS Fixes	GPS-on Time (min)	Average Acquisition Time per Fix (s)	Running Time (min)	Data Sent (bytes)	Data Received (bytes)
30	On-device	12,341	1614	7.84	7790	-	-
	MCC oriented	9324	770	4.98	5402	1,084,901	18,796
60	On-device	10,816	1219	6.76	12,028	-	-
	MCC oriented	7205	764	6.45	7907	838,640	14,696
90	On-device	7868	1178	8.91	13,075	-	-
	MCC oriented	5624	546	5.84	8946	653,833	12,223
120	On-device	5189	809	9.26	11,289	-	-
	MCC oriented	4332	387	5.43	8931	504,012	8838
150	On-device	5576	933	9.94	14,998	-	-
	MCC oriented	4564	452	6.06	11,619	530,764	10,309

**Table 4:** Battery gains of the on-device approach vs MCC oriented solution across time, as observed during experiment two.

Remote Experiment Elapsed Time	Sampling Period (s)				
	30	60	90	120	150
20 %	0.04	0.05	0.03	0.02	0.02
40 %	0.11	0.19	0.11	0.08	0.05
60 %	0.44	0.47	0.34	0.45	0.16
80 %	1.45	1.25	1.04	0.95	0.40
100 %	8.5	9	7.75	5.25	5
